# 

**Published:** 1883-07

**Authors:** 


					﻿Whole Number
CCCCLXII.
July, 1883.
Number 1,
Vol. XLVII.
THE
CHICAGO
MEDICAL
Journals Examiner.
CHICAGO:
PUBLISHED BY THE CHICAGO MEDICAL PRESS ASSOCIATION
188 Clark Street.
(ag“Read the Notice of this Journal among Advertisements.
				

